# Evaluating the effect of immune cells on the outcome of patients with mesothelioma

**DOI:** 10.1038/bjc.2017.269

**Published:** 2017-08-17

**Authors:** Serena J Chee, Maria Lopez, Toby Mellows, Sharmali Gankande, Karwan A Moutasim, Scott Harris, James Clarke, Pandurangan Vijayanand, Gareth J Thomas, Christian H Ottensmeier

**Affiliations:** 1 Cancer Sciences Unit, Faculty of Medicine, University of Southampton, Tremona Road, Southampton SO16 6YD, UK; 2 NIHR Southampton Biomedical Research Centre, Tremona Road Southampton General Hospital Southampton SO16 6YD, UK; 3 Department of Cellular Pathology, University Hospital Southampton NHS Foundation Trust, Tremona Road, Southampton SO16 6YD, UK; 4 Clinical and Experimental Sciences, Southampton National Institute for Health Research Respiratory Biomedical Research Unit, University of Southampton, Faculty of Medicine, Southampton SO16 6YD, UK; 5 Public Health Sciences and Medical Statistics, University of Southampton, Tremona Road, Southampton SO16 6YD, UK; 6 NIHR CRUK Experimental Cancer Medicine Centre Southampton, Tremona Road, Southampton SO16 7YD, UK

**Keywords:** mesothelioma, cancer immunology, tissue microarray

## Abstract

**Background:**

We systematically assessed the prognostic and predictive value of infiltrating adaptive and innate immune cells in a large cohort of patients with advanced mesothelioma.

**Methods:**

A tissue microarray from 302 samples was constructed. Markers of adaptive immune response in T-cells (CD8^+^, FOXP3^+^, CD4^+^, CD45RO^+^, CD3^+^) and B-cells (CD20^+^), and of innate immune response; neutrophils (NP57^+^), natural killer cells (CD56^+^) and macrophages (CD68^+^) were evaluated.

**Results:**

We found that in the epithelioid tumours, high CD4^+^ and CD20^+^ counts, and low FOXP3^+^, CD68^+^ and NP57^+^ counts linked to better outcome. In the non-epithelioid group low CD8^+^ and low FOXP3^+^ counts were beneficial.

On multivariate analysis low FOXP3^+^ remained independently associated with survival in both groups. In the epithelioid group additionally high CD4^+^, high CD20^+^, and low NP57^+^ counts were prognostic.

**Conclusions:**

Our data demonstrate for the first time, in predominately advanced disease, the association of key markers of adaptive and innate immunity with survival and the differential effect of histology. A better understanding of the immunological drivers of the different subtypes of mesothelioma will assist prognostication and disease-specific clinical decision-making.

Mesothelioma is a malignancy most commonly affecting the pleura, but can also arise in the peritoneum, tunica vaginalis and pericardium. It is mainly associated with asbestos exposure with a lag time from exposure to diagnosis of up to 30–40 years (Scherpereel *et al*, 2010). There is also a genetic component, with family clusters of patients who possess BAP1 mutations having an increased likelihood of developing mesothelioma and uveal melanoma (Testa *et al*, 2011).

Mesothelioma incidence is predicted to peak in the United Kingdom in 2020. Worldwide however, the incidence will continue to rise as asbestos is still mined and used in industry in many countries outside of Europe, Australia and the United States. It is not yet clear if inhalation of carbon nanotubes, increasingly used in the fields of electronics and medicine, cause similar sequalae as inhalation of asbestos fibres (Jaurand *et al*, 2009).

Mesothelioma usually presents late, with diagnosis typically made by a combination of radiological findings and pleural biopsy/pleural fluid cytology. The median survival following a diagnosis of mesothelioma is 4–18 months despite treatments like chemotherapy, radiotherapy and surgery.

Asbestos-associated pleural mesothelioma results from chronic inflammation, due to the body’s inability to deal with a foreign antigen in the form of inhaled asbestos fibres. Three histological subtypes of mesothelioma are recognised. Epithelioid mesothelioma carries the best prognosis, sarcomatoid mesothelioma is the most aggressive and tumours with mixed morphology have intermediate outcomes.

The role of the immune system in cancer is well established and is critically involved in immunoediting and surveillance; tumour progression results from immune escape (Dunn *et al*, 2004; Schreiber *et al*, 2011). In many solid tumours, including colorectal, lung, breast and oropharyngeal, tumour infiltrating lymphocytes (TILs) confer a survival benefit (Galon *et al*, 2006; Al Shibli *et al*, 2008; Gooden *et al*, 2011; García-Martínez *et al*, 2014; Ward *et al*, 2014). A meta-analysis of 23 studies of solid tumours demonstrated the presence of CD8^+^ TILs conferred a prognostic advantage and a similar analysis of six studies of CD4^+^ TILs showed a significant effect on overall survival (Gooden *et al*, 2011). A more recent meta-analysis of nearly 18 000 tumours in 39 cancers found higher levels of T-cell fractions to be generally associated with better survival (Gentles *et al*, 2015).

In mesothelioma, the impact of TIL density is less clear. Previous small surgical studies have suggested that tumour CD8^+^ T-cell infiltration is associated with better survival (Anraku *et al*, 2008; Yamada *et al*, 2010). More recently, a larger series demonstrated tumour CD4^+^ T-cell infiltration confers a survival advantage in epithelioid mesothelioma (Uijie *et al*, 2015). These three cohorts consisted mainly of patients who were fit enough to undergo surgical resection.

The link between T-cell density in the tumour and outcome for advanced disease is not resolved. In the study presented here we sought to characterise the link between morphological density of immune cells in mesothelioma and outcome in a predominately treatment naïve cohort. We undertook an assessment of innate and adaptive immune cells in a cohort of 302 patients from a large UK centre, using immunohistochemical evaluation of immune cells.

Establishing which immune cells are associated with clinical outcome may also have a bearing on the evaluation of response to treatments such as chemotherapy and immunotherapy. Immunotherapy trials in mesothelioma are underway with checkpoint inhibitors such as tremelimumab targeting CTLA4 or pembrolizumab directed against PD-1 (Calabrò *et al*, 2013; Alley *et al*, 2017). Similarly, targeting WT1 (Zauderer, 2010) and mesothelin (Kelly *et al*, 2012; Hassan *et al*, 2015) by vaccination or CAR (Rosenberg, 2012) therapies is an area of intense interest. To date, the factors that are associated with treatment success are not yet fully understood. Where data regarding chemotherapy was available, we evaluated whether particular immune cells were associated with treatment success.

## Materials and methods

### Case selection

Ethical approval for this study (NRES Southampton and South West Hampshire LREC 10/H0504/32) was in place at our institution.

Patients included in the cohort had a pathological diagnosis of mesothelioma dated at least 2 years prior to analysis. A total of 302 consecutive formalin fixed paraffin embedded tissue blocks archived between 2004 and 2012 were assessed by haematoxylin-eosin staining to ensure they contained sufficient tissue for further immunohistochemical evaluation. A tissue microarray (TMA) was generated from this cohort. Some cases could not be evaluated as there was tissue loss in the formation of the TMA. Cases were included in the analysis if at least three high-powered fields were available. A subcohort of ∼170 cases was evaluated for CD3^+^ T-cells and CD56^+^ natural killer cells. All samples were taken at the time of diagnostic biopsy or surgical intervention when no systemic treatment had been given.

Clinical data were collected from patient records. Where survival data were not available locally, the date and cause of death was retrieved from the national cancer registry (Public Health England) following a formal data-access application.

Post diagnosis treatment data were available from 166 patients. Of these 61 (37%) underwent chemotherapy and 105 (63%) did not. Treatment data were not available for 136 patients who had been referred to our centre for diagnostic procedures but were then managed at other sites (Table 1).

Of the 302 patients, 259 (86%) underwent palliative symptom management with thoracoscopy and pleural biopsy followed by talc pleurodesis; and 7 (2%) underwent image guided pleural biopsy. Thirty-six (12%) underwent a radical surgical approach including decortication/pleurectomy/pleuro-pneumonectomy (Table 1).

Patients were excluded from the analysis based on the following criteria: tissue loss resulting in availability of less than three high-powered fields, unavailability of survival data, death within 30 days of biopsy, lack of treatment data in the post diagnosis treatment group and an undefined histological morphology.

### Histopathological analysis–tissue microarray

Tumour histology was re-reviewed by pathologists GJT, KAM and SG. Haematoxylin-eosin stained slides from all available cases were assessed and three representative areas of tumour selected.

Triplicate 1 mm cores were taken from the corresponding formalin fixed paraffin embedded tissue block using a semi-automated system (Aphelys Minicore 2, Mitogen, Harpenden, UK) to generate a TMA. TMA sections (4 *μ*m) were used for haematoxylin-eosin and immunohistochemical staining.

Immunohistochemistry was performed using an automated platform (Dako Autostainer) in a CPA-accredited clinical cellular pathology department using antibodies optimised to national diagnostic standards (NEQAS). The antibodies used were anti-human CD3 1:200 (clone F7.2.38; Dako, Carpinteria, CA, USA), anti-human CD4 1:50 (clone 4B, Dako), CD8 RTU (Clone C8/14 4B, Dako), CD20 1:250 (Clone L26, Dako), CD45RO 1:2500 (Clone UCHL-1, Dako), CD56 RTU (Clone 123C3, Dako), NP57 1:100 (Dako), CD68 RTU (Clone PG-M1, Dako), Wilms Tumour 1 RTU (Clone 6F-H2, Dako), FoxP3 1:100 (Clone 236A/E7, Abcam, Cambridge, UK).

Triplicate random high-power fields (× 400) were counted manually per core across three cores on the Olympus dotSlide (SC). The average of 3–9 counts per patient was calculated to allow for intra-tumoural heterogeneity. The variability in the number of counts per patient relates to the number of viable cores present on the TMA.

**Figure 1:**
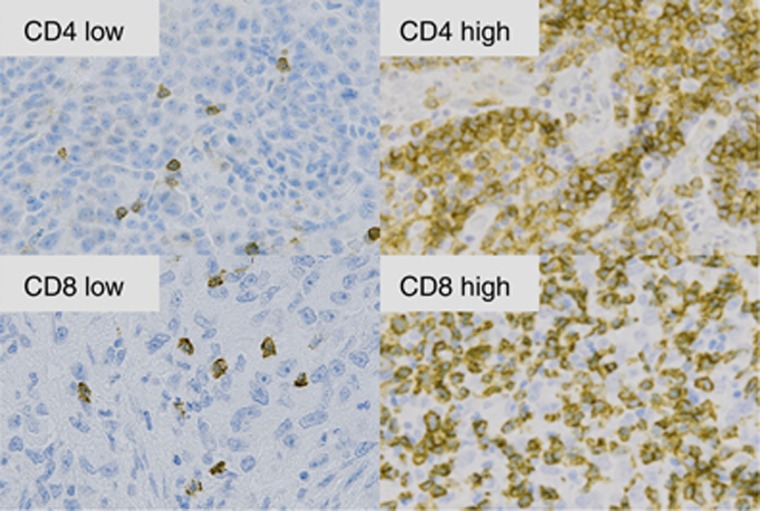
**Representative low and high T-cell densities (× 400) magnification: CD4^+^ and CD8^+^ cells.**

The mean of each antibody count was taken as the cutoff point between high and low counts. Representative high and low T-cell densities are shown in Figure 1.

### Statistical analysis

Statistical analyses were performed using SPSS Version 22 (IBM Corp. Released 2013. IBM SPSS Statistics for Windows, Version 22.0, IBM Corp., Armonk, NY, USA). Survival time was measured from the time of diagnosis to the time of death. Patients for whom the date of death was unknown were censored from the survival analyses. Kaplan–Meier plots with log-rank tests, univariate and multivariate Cox proportional hazard models were used to analyse the survival data. Separate subtype models were fitted due to the presence of interactions between subtype and several of the immune markers analysed. A *P*-value of <0.05 was considered significant.

Our cohort was divided into epithelioid and non-epithelioid (biphasic and sarcomatoid) mesothelioma subtypes for the purposes of analysis as there were only 41 cases of sarcomatoid mesothelioma.

## Results

Clinical and demographic characteristics of our patient cohort are shown in Table 1. The median age was 72 (41–90) years, 81% of the patients were men. Of the 302 tumours, 172 were classified as epithelioid (57%), 82 as biphasic (27%) and 41 as sarcomatoid (14%) subtypes. Seven cases (2%) had an undefined morphology. The median follow-up was 278 days (9.3 months) and the minimum follow-up time was 30 days. There were 293 deaths from mesothelioma over the study period. Survival analysis by histological subtype was consistent with published data. The median overall survival for epithelioid mesothelioma in our cohort was 342 days (11.4 months) (95% CI 277–406 days) and for non-epithelioid mesothelioma was 205 days (6.8 months) (95% CI 146–260 days) (Table 1).

The adaptive immune response, particularly involving cytotoxic T-cells, has been shown to influence the prognosis of many tumour types. We examined expression of markers for cytotoxic T-cells (CD8^+^), regulatory T-cells (FOXP3^+^), helper T-cells (CD4^+^), memory T-cells (CD45RO^+^), T-cells (CD3^+^) and B-cells (CD20^+^) in the epithelioid and non-epithelioid subtypes.

The mean count/high-power field was used to define the cut point between high and low values per marker evaluated. Median overall survival with 95% confidence interval and *P*-values for the epithelioid and non-epithelioid groups are shown in Table 2.

**Figure 2:**
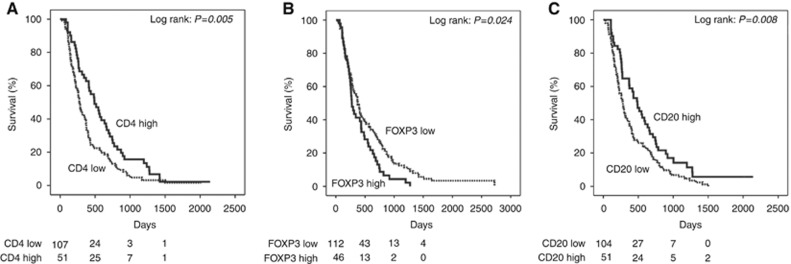
**Kaplan–Meier survival curves of adaptive immune markers associated with survival in epithelioid mesothelioma.** (**A**) Kaplan–Meier curves for epithelioid mesothelioma survival according to CD4^+^ T-cell counts (log-rank test, *P*=0.005). (**B**) Kaplan–Meier curves for epithelioid mesothelioma survival according to FOXP3^+^ T-regulatory cell counts (log-rank test, *P*=0.024). (**C**) Kaplan–Meier curves for epithelioid mesothelioma survival according to CD20^+^ B-cell counts (log-rank test, *P*=0.008).

In the epithelioid group, we found that high CD4^+^ (*P*=0.005), low FOXP3^+^ (*P*=0.024) and high CD20^+^ counts (*P*=0.008) were associated with a better outcome. The relevant Kaplan–Meier curves are shown in Figure 2A–C. These markers were associated with a longer overall survival of 7, 3 and 7 months respectively compared to the CD4^+^ low, FOXP3^+^ high and CD20^+^ low groups. CD8^+^ T-cell counts were not associated with survival in the epithelioid group (*P*=0.983).

**Figure 3:**
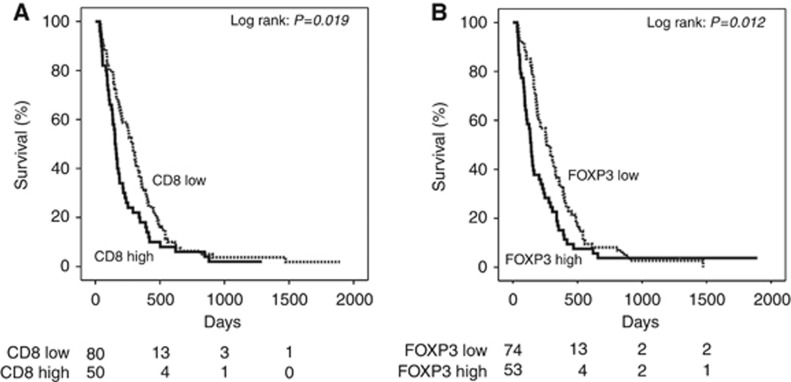
**Kaplan–Meier survival curves of adaptive immune markers associated with survival in non-epithelioid mesothelioma.** (**A**) Kaplan–Meier curves for non-epithelioid mesothelioma survival according to CD8^+^ T-cell counts (log-rank test, *P*=0.019). (**B**) Kaplan–Meier curves for non-epithelioid mesothelioma survival according to FOXP3^+^ T-regulatory cell counts (log-rank test, *P*=0.012).

In the non-epithelioid group, positive prognostic markers were low CD8^+^ (*P*=0.019) and low FOXP3^+^ (*P*=0.012) T-cell counts. Kaplan–Meier curves are shown in Figure 3A and B. Both markers were associated with a 4-month longer overall survival in the non-epithelioid group compared to the CD8^+^ high and FOXP3^+^ high groups.

The tumour microenvironment also contains innate immune cells and we evaluated the expression of markers of neutrophils (NP57^+^), natural killer cells (CD56^+^) and macrophages (CD68^+^).

In the epithelioid group, we found the positive predictors of outcome from innate immune cells were low CD68^+^ (*P*=0.026) and low NP57^+^ (*P*=0.006) counts. Kaplan–Meier curves are shown in Supplementary Figure 1A and B. These markers were both associated with a 3-month longer overall survival in the epithelioid group compared to the CD68^+^ high and NP57^+^ high groups. In the non-epithelioid group, none of the markers of innate immunity tested were significantly associated with survival.

Wilms tumour 1 (WT1) is expressed in most cases of mesothelioma. Expression was not associated with differential survival in either morphology in our data set.

Multivariate analysis was performed next based on the parameters that were significant on univariate analysis. In the epithelioid group, a high CD4^+^ (*P*=0.003), high CD20^+^ (*P*=0.010), low FOXP3^+^ (*P*=0.000414) and low NP57^+^ (*P*=0.038) counts remained significantly associated with survival. In the non-epithelioid group, a low number of FOXP3^+^ cells associated with survival, *P*=0.043. The data for the multivariate analysis of both groups is shown in Table 3.

The ratio of CD4^+^/CD8^+^ with a cut point of 1 between low and high counts was analysed. A ratio of >1 was associated with longer survival only in the epithelioid group, 99 low, 59 high (158 total) *P*=0.047. Kaplan–Meier curves are shown in Supplementary Figure 2A and B.

Of 166 patients managed in our own centre, 61 had received chemotherapy. These 61 chemotherapy-treated patients had a significantly better survival than those who were not given chemotherapy (*P*<0.0001), likely reflecting both patient selection and effect of treatment. In the 61 patients treated with chemotherapy, a high CD4^+^ count identified patients who lived longer (*P*=0.034); no effect was seen for CD8^+^ T-cells.

Of 94 patients with epithelioid histology, 42 were treated with chemotherapy. Chemotherapy administration but also high CD4^+^, high CD20^+^, low FOXP3^+^ counts were independent prognostic factors (*P*=0.001, *P*=0.005, *P*=0.015, *P*=0.046) in multivariate analysis. In the group of 72 non-epithelioid cases, 19 patients had been treated with chemotherapy; only chemotherapy administration was independently associated with survival (*P*=0.029).

## Discussion

We have evaluated a large unselected cohort of patients for evidence of whether immune attack occurs in mesothelioma and how this might affect survival. To our knowledge, this is the largest cohort of mesothelioma containing all subtypes analysed to date. Our data represent a patient population in whom treatment was given with palliative intent from the outset (88%). Only 37% of the cohort received palliative chemotherapy and our cohort is clinically distinct from published data sets that have evaluated TIL density in mesothelioma to date and where the focus has been on operable disease (Anraku *et al*, 2008; Yamada *et al*, 2010; Uijie *et al*, 2015).

We studied markers of adaptive (CD3^+^, CD4^+^, CD8^+^, T regulatory, T memory and B-cells) and innate immunity (macrophages, natural killer cells and neutrophils). Epithelioid and non-epithelioid mesothelioma are clinically distinct diseases, and our data demonstrate they are immunologically different also; adaptive immune cell infiltrates differentially link to outcome between subtypes.

A limitation to this study is the analysis is based on staining small biopsy specimens in the TMA which may not be representative of the whole tumour. This is important as mesothelioma has been shown to be polyclonal in origin (Comertpay *et al*, 2014). We attempted to correct for inter-tumoural heterogeneity by taking three cores across the tumour and 3–9 counts per patient.

Unexpectedly, high CD8^+^ density appears not to be beneficial for survival in mesothelioma patients. In the epithelioid group the CD8^+^ T-cell density was indifferent for outcome; in the non-epithelioid group, a low number of CD8^+^ T-cells linked with a survival advantage, although this observation was not maintained in the multivariate analysis. This is in contrast to observations in many other solid cancers where a high density of CD8^+^ TILs has been shown to confer a survival advantage (Galon *et al*, 2006; Al Shibli *et al*, 2008; Gooden *et al*, 2011; García-Martínez *et al* 2014; Ward *et al*, 2014). Published data suggest that non-epithelioid mesothelioma is more likely to be PD-L1 positive and to have a higher proportion of proliferating CD8^+^ T-cells (Awad *et al*, 2016). This suggests that in our data the presence of CD8^+^ T-cells is also likely to be associated with high PD-L1 expression. It is unclear whether such PDL1 expression in the tumour cells is in part or even mostly a reflection of immune attack via IFN signalling from immune cells (and therefore reflects immunogenicity of the cancer tissue) or conversely whether PDL1 expression might be driven by another pathway in mesothelioma, with only deleterious effects on T-cell responses.

A high density of CD4^+^ TILs conferred a survival advantage in the epithelioid group, both on univariate and multivariate analysis. CD4^+^ T-cells are important for activating a range of tumour-reactive immune cells including CD8^+^ T-cells and B-cells (Ding *et al*, 2010). Further work to establish the targets of CD4^+^ T-cell recognition is needed to better understand the balance of effector and regulatory CD4^+^ T-cells in mesothelioma. This would then also allow a linkage to mutational status and immune attack and targeting of such mutations, for example by vaccination.

A key CD4^+^ T-cell population that limits the function of effector and helper T-cells are FOXP3^+^CD4^+^ regulatory T-cells. Consistent with the current understanding of their role, we observe that a high number of FOXP3^+^ T-cells was significantly associated with poorer survival in both morphological subgroups of mesothelioma. The effect of FOXP3^+^ T-reg likely has disease-specific features. While in many solid tumour types a lower T-reg density is good for the patient (Shang *et al*, 2015), there are some exceptions, such as head and neck squamous cell carcinoma, where higher FOXP3^+^ T-cell density is linked to better locoregional control (Badoual *et al*, 2006). It may simply be though that this apparent contradiction reflects a parallel influx of protective and suppressive immune cells, as in head and neck cancer where globally the number of immune cells is tightly linked to survival (Ward *et al*, 2014; Wood *et al*, 2016).

We evaluated, whether the sensitivity of quantification of the immune cell infiltrate could be improved by assessing the relative density of CD4^+^ and CD8^+^ T-cells. An easy way of expressing this is by calculating the ratio of CD4^+^/CD8^+^ T-cells, a measure that then becomes independent of the absolute abundance of the T-cells in the tissue. A high CD4^+^/CD8^+^ ratio has been previously evaluated in different types of cancer and was found to be associated with a good outcome in cervical squamous cell carcinoma and a poor outcome in colorectal cancer (Diederichsen *et al*, 2003; Shah *et al*, 2011). In our cohort, a CD4^+^/CD8^+^ ratio of greater than 1 was associated with better survival in the overall and epithelioid groups and this parameter warrants further analysis in other cancers to determine if it might be a useful prognostic marker for survival.

There is increasing recognition of the important role B-cells play in adaptive immune attack in the tumour microenvironment. Tumour-associated B-cells induce and regulate T-cell immune responses through antigen presentation and CD4^+^ T-cell activation, contributing to the differentiation of CD4^+^ T-cells and polarisation of Th1 and Th2 subsets (Lund and Randall, 2010; Baumgarth, 2011; Bao *et al* 2014). In our cohort, a high density of CD20^+^ in the epithelioid group was associated with better survival. This remained significant on multivariate analysis and our data are consistent with Ujiie *et al* (2015). Similar to T-cells, there is variation in the prognostic effect of B-cell infiltration between tumour types. In colorectal cancer, an increased B-cell gene expression was significantly associated with survival (Bindea *et al*, 2013). Our own data in head and neck squamous cell carcinoma has demonstrated a high B-cell infiltrate to be associated with better outcome (Wood *et al*, 2016). It is unclear however what the B-cells might contribute immunologically, and whether are simply drawn to the tumour in response to cytokine stimulation.

Tumour-associated inflammation is recognised as a key hallmark of cancer (Grivennikov *et al*, 2010; Hanahan and Weinberg, 2011) and innate immune cells such as macrophages and neutrophils form an important and complex part of the tumour microenvironment. Tumour-associated macrophages can adopt a pro-tumourigenic role by promotion of angiogenesis and metastases and preventing T-cell attack on tumour cells and once the tumour is established, the macrophages are polarised to a pro-tumour phenotype (Noy and Pollard, 2014). In our cohort, a low CD68^+^ count was significant in the epithelioid group, similar to findings by Ujiie *et al* (2015), but not in the non-epithelioid group. The association of a low infiltration of tumour-associated macrophages and survival is consistent with the theory of chronic asbestos-induced inflammation driving the development of mesothelioma in a process of ‘frustrated phagocytosis’ (Bograd *et al*, 2011).

Tumour-associated neutrophils similarly can adopt a pro-tumourigenic role by involvement in angiogenesis and creating a pro-invasive and pro-metastatic environment and may also play an anti-tumourigenic role (Hanahan and Coussens, 2012). Consistent with this, in our cohort, a low number of NP57 cells were associated with better survival in the epithelioid group. To our knowledge, our group is the first to detect the prognostic value of a low neutrophil count on survival in epithelioid mesothelioma.

Beyond histological typing into epithelioid, sarcomatoid and mixed types, markers that predict response to treatment in mesothelioma are lacking. In other solid tumours such as breast, rectal and oesophageal cancer immunological features that predict for pathological complete response and survival following chemotherapy have been examined. In a breast cancer cohort, a pre-treatment high CD4^+^/CD8^+^ ratio was an independent predictor of pathological complete response after neo-adjuvant chemotherapy and was associated with better prognosis (García-Martínez *et al*, 2014) as was the presence of CD20^+^ B-cells (Brown *et al*, 2014). We therefore wondered if TIL density would link to treatment response in mesothelioma. We found that this was the case: in 61 patients who had received chemotherapy CD4^+^ T-cell high cases identified patients who had a better outcome. This remained true also if only the 42 patients with epithelioid histology and who received chemotherapy were considered and suggest that CD4^+^ T-cells may also have a predictive value in mesothelioma.

In summary, our data demonstrate for the first time an association of survival with high CD4^+^, low FOXP3^+^, high CD20^+^, low NP57^+^ and low CD68^+^ counts in epithelioid mesothelioma, treated with palliative intent. Low CD8^+^ and low FOXP3^+^ T-cell densities emerge as prognostic in non-epithelioid mesothelioma. Given that epithelioid and non-epithelioid mesothelioma behave very differently clinically, the underpinning differences in potential immunological drivers of the different subtypes are intriguing and warrant further study and will benefit from mapping onto the emerging stratifiers using genomic analyses (Bueno *et al*, 2016). Emerging data suggesting that sarcomatoid mesothelioma may be more responsive to immunotherapy than the epithelioid subtype (Mansfield *et al*, 2014; Cedrés *et al*, 2015), but more work is needed to understand the reason behind this difference. Our data illustrate that the morphological differences, linked to outcome also find their reflection in the adaptive and innate immune events that are present in the cancer. Their functional understanding will open the door towards rational targeting of immune pathways to improve the outcomes of patients with this devastating disease.

## Figures and Tables

**Table 1:** Demographics of patient cohort

**Variable**	***N*** **(%)**	**Median OS days (months)**	**95% CI**	***P*****-value**
**Overall group**	302 (100%)	278 (9.2)	247–308	
Age				
<65	63 (21%)	349 (11.6)	257–440	0.157
⩾65	239 (79%)	263 (8.7)	229–296	
Sex				
Male	243(81%)	275 (9.2)	243–306	0.682
Female	59 (19%)	291 (9.7)	194–387	
Subtype				
Epithelioid	172 (57%)	342 (11.4)	277–406	**<0.0001**
Non-epithelioid	130 (43%)	205 (6.8)	146–260	
Biphasic	82 (27%)	216 (7.2)	152 to279	
Sarcomatoid	41 (14%)	187 (6.2)	112 to261	
Undefined	7 (2%)			
**Intervention**				
Chemotherapy status known				
Overall	166	280 (9.3)	239–320	**<0.0001**
Yes	61 (37%)	460 (15.3)	360–559	
No	105 (63%)	205 (6.8)	159–250	
Chemotherapy status unknown		136		
Surgery				
Overall	295	279 (9.3)	247–310	0.735
Radical	36 (12%)	330 (11.0)	185–474	
Palliative	259 (86%)	274 (9.1)	242–305	
No surgery		7(2%)		

Abbreviations: OS=overall survival; CI=confidence interval.

Significant log rank *P*-value<0.05. Bold values indicate statistical significance.

**Table 2:** Univariate analysis of overall survival and immune parameters

**Marker**	**Mean counts/high powered field**	***N*** **(%)**	**Median OS (months)**	**95% CI**	***P*****-value**
**Adaptive**					
CD4					
Epithelioid		158	319 (10.6)	250–387	**0.005**
	Low<15.1	107 (68%)	275 (9.2)	240–309	
	High>15.1	51 (32%)	490 (16.3)	350–629	
Non-epithelioid		126	205 (6.8)	150–259	0.383
	Low<15.1	87 (69%)	212 (7.1)	145–278	
	High>15.1	39 (31%)	185 (6.2)	117–252	
CD8					
Epithelioid		172	342 (11.4)	277–406	0.983
	Low<20.1	123 (72%)	342 (11.4)	264–419	
	High>20.1	49 (28%)	373 (12.4)	186–559	
Non-epithelioid		130	205 (6.8)	149–260	**0.019**
	Low<20.1	80 (62%)	278 (9.3)	228–327	
	High>20.1	50 (38%)	152 (5.1)	130–173	
CD45RO					
Epithelioid		159	342 (11.4)	276–407	0.339
	Low<22	121 (76%)	342 (11.4)	275–408	
	High>22	38 (24%)	296 (9.9)	81–510	
Non-epithelioid		128	203 (6.8)	147–258	0.105
	Low<22	77 (60%)	252 (8.4)	176–327	
	High>22	51 (40%)	164 (5.5)	129–198	
FOX P3					
Epithelioid		158	369 (12.3)	285–452	**0.024**
	Low<4	112 (71%)	374 (12.5)	314–433	
	High>4	46 (29%)	272 (9.1)	214–329	
Non-epithelioid		127	205 (6.8)	146–263	**0.012**
	Low<4	74 (58%)	261 (8.8)	173–348	
	High>4	53 (42%)	141 (4.7)	116–165	
CD20					
Epithelioid		155	313 (10.4)	245–380	**0.008**
	Low<15	104 (67%)	279 (9.3)	220–337	
	High>15	51 (33%)	488 (16.2)	358–617	
Non-epithelioid		125	197 (6.6)	147–246	0.227
	Low<15	104 (83%)	205 (6.8)	141–268	
	High>15	21 (17%)	185 (6.2)	117–252	
CD3					
Epithelioid		84	410 (13.7)	348–471	0.387
	Low<34	61 (73%)	373 (12.4)	287–458	
	High>34	23 (27%)	490 (16.3)	305–674	
Non-epithelioid		86	170 (5.7)	124–215	0.123
	Low<34	48 (56%)	170 (5.7)	102–237	
	High>34	38 (44%)	169 (5.6)	126–211	
**Innate**					
CD68					
Epithelioid		164	341 (11.4)	275–406	**0.026**
	Low<27	111 (68%)	373 (12.4)	277–468	
	High>27	53 (32%)	284 (9.5)	200–367	
Non-epithelioid		126	205 (6.8)	149–260	0.927
	Low<27	62 (49%)	247 (8.2)	175–318	
	High>27	64 (51%)	164 (5.5)	98–229	
NP57					
Epithelioid		158	313 (10.4)	242–383	**0.006**
	Low<1.6	129 (82%)	356 (11.9)	264–447	
	High>1.6	29 (18%)	272 (9.1)	202–341	
Non-epithelioid		120	197 (6.6)	150–243	0.291
	Low<1.6	104 (87%)	203 (6.8)	145–260	
	High>1.6	16 (13%)	164 (5.5)	105–222	
**CD56**					
Epithelioid		89	426 (14.2)	364–487	0.786
	Low<3	69 (78%)	410 (13.7)	342–477	
	High>3	20 (22%)	438 (14.6)	96–779	
Non-epithelioid		83	177 (5.9)	132–221	0.239
	Low<3	61 (73%)	169 (5.6)	138–199	
	High>3	22 (27%)	247 (8.2)	130–363	
**Other**					
WT1					
Epithelioid		160	319 (10.6)	246–391	0.802
	Low<42	68 (42%)	319 (10.6)	232–405	
	High>42	92 (58%)	312 (10.4)	203–420	
Non-epithelioid		126	197 (6.6)	143–250	0.875
	Low<42	102 (81%)	185 (6.2)	135–234	
	High>42	24 (19%)	212 (7.1)	151–272	

Abbreviations: OS=overall survival; CI=confidence interval.

Significant log rank *P* value<0.05. Bold values indicate statistical significance.

**Table 3:** Multivariate analysis of overall survival in whole cohort

	**Marker**	**HR**	**95% CI**	***P*****-value**
	CD4	0.499	0.313–0.795	**0.003**
	FOX P3	2.399	1.476–3.900	**<0.001**
Epithelioid *n*=131	CD20	0.560	0.361–0.869	**0.010**
	CD8	0.939	0.579–1.522	0.797
	CD68	1.325	0.909–1.930	0.143
	NP 57	1.595	1.026–2.481	**0.038**
	CD4	0.800	0.465–1.375	0.420
	FOX P3	1.554	1.015–2.381	**0.043**
Non-epithelioid *n*=111	CD20	1.122	0.619–2.034	0.704
	CD8	1.470	0.939–2.302	0.092
	CD68	0.961	0.646–1.429	0.843
	NP 57	1.338	0.769–2.325	0.303

Abbreviations: HR=hazard Ratio; CI=confidence interval.

Cox proportional hazards regression model, significant *P* value<0.05. Bold values indicate statistical significance.
