# Supplementary figures and images for: Evaluating the effect of immune cells on the outcome of patients with mesothelioma
Source: Br J Cancer. 2017 Aug 17;117(9):1341–8. doi: 10.1038/bjc.2017.269 (PMC5672927; doi:10.1038/bjc.2017.269)

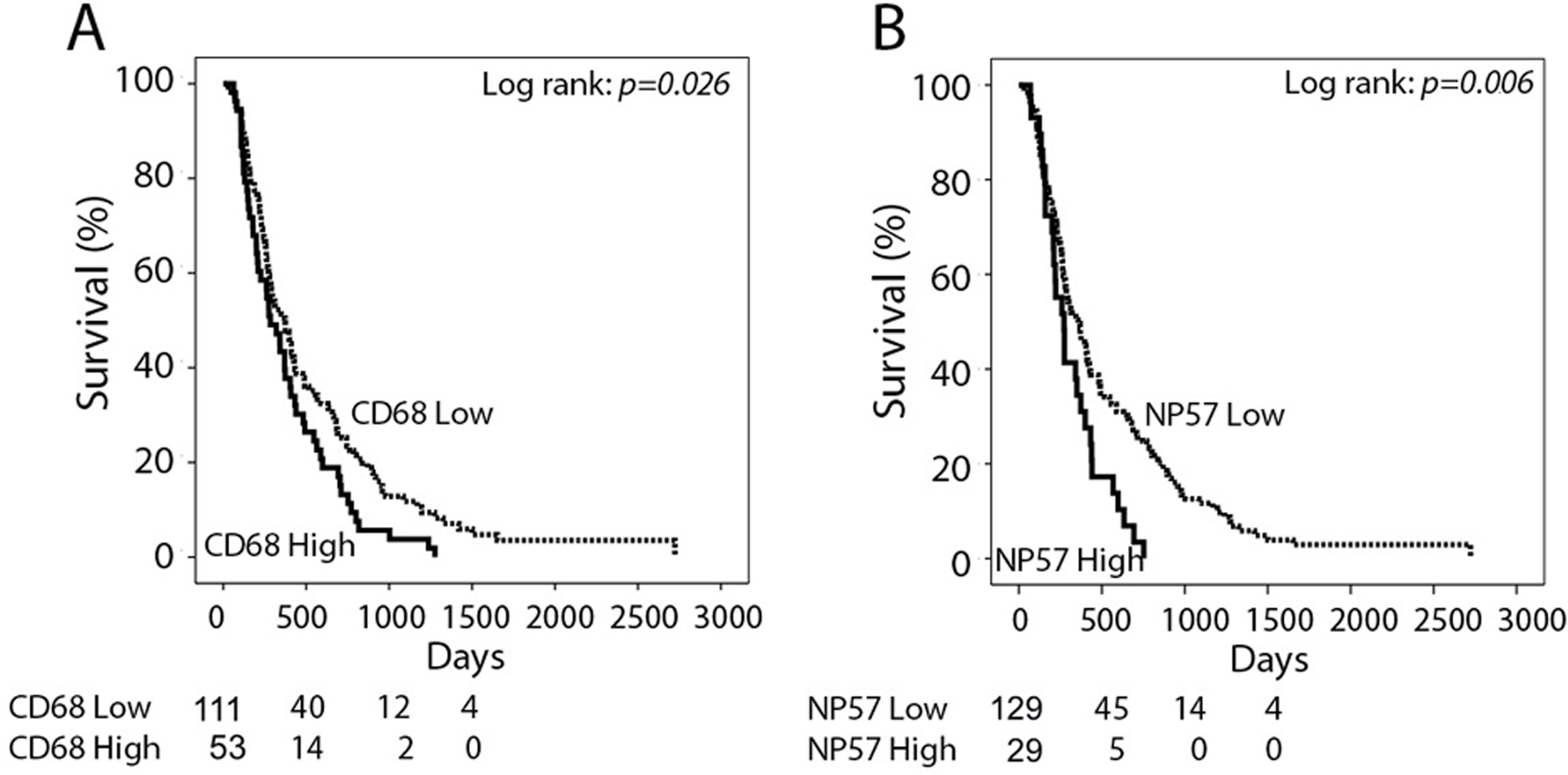

Supplement: Supplementary Figure 1 [file bjc2017269x1.tif]

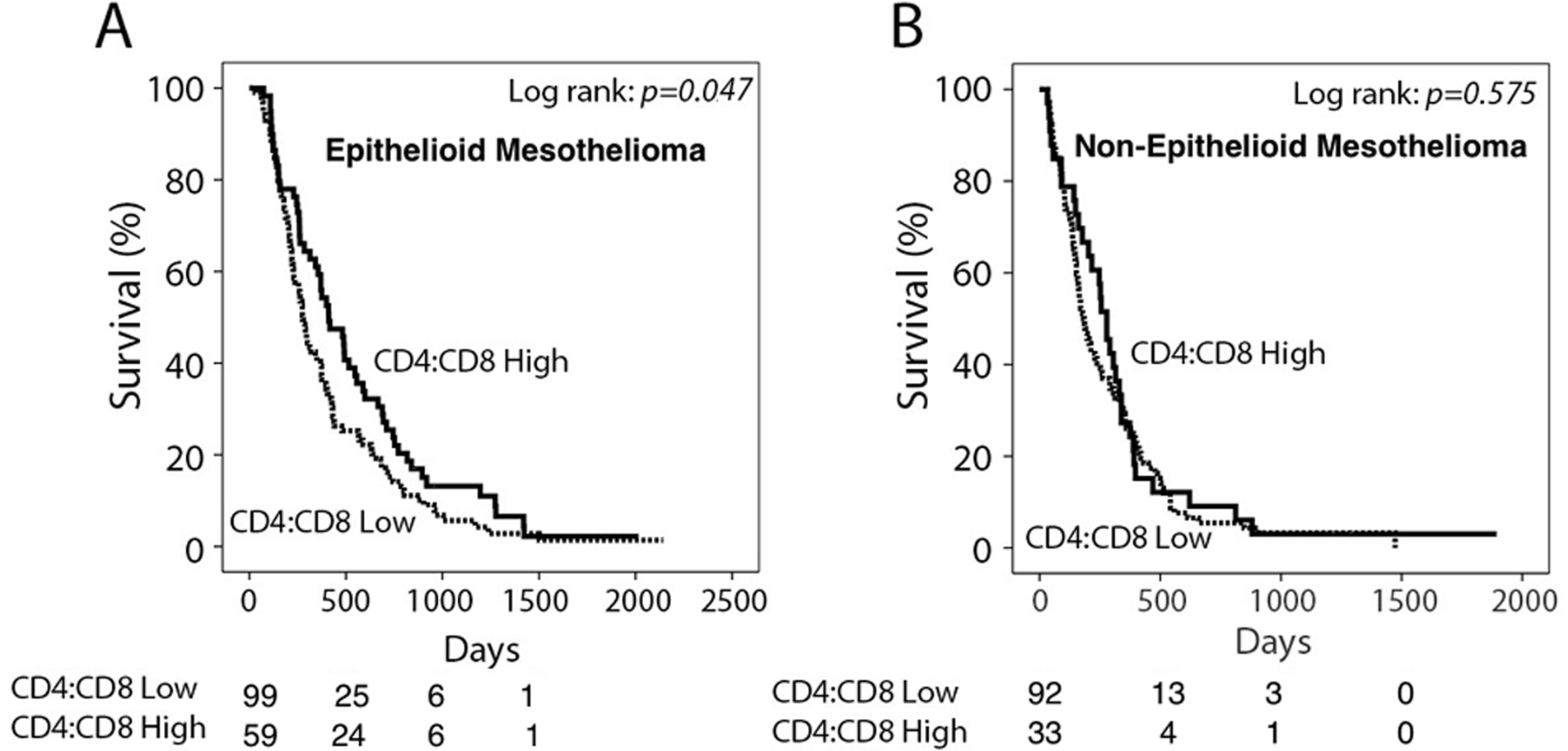

Supplement: Supplementary Figure 2 [file bjc2017269x2.tif]
